# PD-L1 Inhibits T Cell-Induced Cytokines and Hyaluronan Expression *via* the CD40-CD40L Pathway in Orbital Fibroblasts From Patients With Thyroid Associated Ophthalmopathy

**DOI:** 10.3389/fimmu.2022.849480

**Published:** 2022-05-10

**Authors:** Zhibin Liu, Yao Liu, Mingming Liu, Qingjia Gong, Anjie Shi, Xiuhong Li, Xu Bai, Xiaoyue Guan, Bing Hao, Feila Liu, Xing Zhou, Hongfeng Yuan

**Affiliations:** ^1^Department of Ophthalmology, Daping Hospital, Army Medical University, Chongqing, China; ^2^Department of Ortibal Surgery, Chongqing Aier Hospital, Chongqing, China; ^3^School of Pharmacy and Bioengineering, Chongqing University of Technology, Chongqing, China; ^4^Department of Pharmacy, Chongqing Hospital of Traditional Chinese Medicine, Chongqing, China; ^5^Chongqing Key Laboratory of Medicinal Chemistry & Molecular Pharmacology, Chongqing University of Technology, Chongqing, China

**Keywords:** PD-L1, T cell, orbital fibroblast, CD40, thyroid associated ophthalmopathy

## Abstract

Thyroid associated ophthalmopathy (TAO), characterized by T cell infiltration and orbital fibroblast activation, is an organ-specific autoimmune disease which is still short of effective and safety therapeutic drugs. The PD-1/PD-L1 pathway has been reported hindering the progression of Graves’ disease to some extent by inhibiting T cell activity, and tumor therapy with a PD-1 inhibitor caused some adverse effects similar to the symptoms of TAO. These findings suggest that the PD-1/PD-L1 pathway may be associated with the pathogenesis of TAO. However, it remains unknown whether the PD-1/PD-L1 pathway is involved in orbital fibroblast activation. Here, we show that orbital fibroblasts from patients with TAO do not express PD-L1. Based on *in vitro* OF-T cell co-culture system, exogenous PD-L1 weakens T cell-induced orbital fibroblast activation by inhibiting T cell activity, resulting in reduced production of sICAM-1, IL-6, IL-8, and hyaluronan. Additionally, exogenous PD-L1 treatment also inhibits the expression of CD40 and the phosphorylation levels of MAPK and NF-κB pathways in orbital fibroblasts of the OF-T cell co-culture system. Knocking down CD40 with CD40 siRNA or down-regulating the phosphorylation levels of MAPK and NF-κB pathways with SB203580, PD98059, SP600125, and PDTC can both reduce the expression of these cytokines and hyaluronan. Our study demonstrates that the orbital immune tolerance deficiency caused by the lack of PD-L1 in orbital fibroblasts may be one of the causes for the active orbital inflammation in TAO patients, and the utilization of exogenous PD-L1 to reconstruct the orbital immune tolerance microenvironment may be a potential treatment strategy for TAO.

## Introduction

Thyroid associated ophthalmopathy (TAO), also designated as Graves’ ophthalmopathy (GO), is a prevalent organ-specific autoimmune disease that has a deteriorating effect on patients’ appearance and vision. TAO most often occurs in association with thyroid disease, such as hyperthyroid and hypothyroid. It is most frequently found complicating Graves’ disease (GD), with a prevalence of more than ninety percent (1–3). Currently, it is difficult to achieve desired results for the treatment of TAO. Corticosteroids, orbital irradiation and surgical decompression have been the mainstay of therapeutic strategies (4–6). However, current medical therapies have limited efficacy, and they are often associated with side effects and other safety concerns (4, 5, 7). In recent years, breakthroughs have been made in the research of immunosuppressants for TAO. Rituximab, Teprotumumab, Tocilizumab, Thalidomide and other drugs, which have been used in the treatment of TAO, partially improve proptosis, diplopia and orbital inflammation of TAO patients (4, 5, 8–10). Due to the insufficient experiences in clinical application, however, the efficacy and safety profile of these drugs remains to be further studied and fully elucidated (4, 5, 8–10). The pathogenesis of TAO is still incompletely understood, which is the result of cellular immunity and humoral immunity under the influence of genetic, environmental and other factors (1–3). A large number of inflammatory cells, mainly T cells, and a few B cells, plasma cells, neutrophils, macrophages, etc., exist in the orbital adipose tissue and the intermuscular space of extraocular muscles from TAO patients (11). T cell, which plays important roles in antigen recognition, orbital fibroblast (OF) activation, B cell differentiation, cytokines release, and hyaluronan (HA) aggregation, is the crucial immune cell mediating orbital inflammation, adipose hyperplasia and extraocular muscle fibrosis in TAO patients (3, 11, 12). Therefore, it is essential to inhibit T cell activity for the treatment of TAO. However, there is still a lack of effective T cell-targeted drugs for the treatment of TAO, which leaves a big gap for the research of TAO therapy.

Recent researches have unveiled a close association between GD and the PD-1/PD-L1 pathway. Programmed cell death-1 (PD-1, or CD279), is a cell surface receptor that functions as an immune checkpoint and plays a crucial role in modulating T cell exhaustion. And its ligand, programmed death-ligand 1 (PD‐L1, or CD274), is mainly expressed on various tumor cells (13, 14). PD-1/PD-L1 is the most crucial negative costimulatory pathway in the immune tolerance of tumor cells (14). The immunosuppression of the PD-1/PD-L1 pathway can attenuate T cell activity, accordingly helping tumor cells escape the immune attack, which plays an important role in promoting the progression of tumor (15, 16). In recent years, PD-1/PD-L1 inhibitors are studied extensively and a few of them have been approved for the treatment of certain types of tumors (17, 18). However, it has also been reported that tumor treatment by inhibiting the PD-1/PD-L1 signaling pathway was likely to cause immune imbalance, resulting in autoimmune diseases, such as multiple sclerosis, inflammatory bowel disease, Hashimoto’s thyroiditis, and rheumatoid arthritis (19, 20). More importantly, the application of a PD-1 inhibitor presented clinical symptoms of TAO, including exophthalmos and enlargement of extraocular muscles (21). Interestingly, the PD-1/PD-L1 pathway is also operative in the pathogenesis of GD and may be a compensatory mechanism to restrain the autoimmune system but probably not to the extent of hindering the progression of GD (22–25). Due to the absence of research about the PD-1/PD-L1 pathway in TAO, however, the role of the PD-1/PD-L1 pathway in the pathogenesis of TAO remains unclear.

In this study, we initially performed an investigation and it was found that OFs did not express PD-L1. This suggested that the orbital immune tolerance deficiency caused by the lack of PD-L1 in OFs may be involved in the pathogenesis of TAO. Then, we constructed a co-culture system of OFs and T cells, utilizing exogenous PD-L1 in an attempt to simulate the orbital immune tolerance microenvironment and discover a potential TAO therapeutic strategy targeting T cell. Additionally, we have also studied the specific mechanism of T cell-induced OF activation *via* the CD40-CD40L pathway.

## Materials and Methods

### Specimen Procurement

From July 2020 to October 2021, 13 subjects, including 8 patients with active TAO (CAS ≥ 3; CAS: clinical activity score) and 5 negative controls (healthy subjects without any known ophthalmopathy or other disease), as **Tables S1**, **S2** showed were recruited from Daping Hospital, Army Medical University (Chongqing, China). Informed consent was obtained from each subject. The study protocol was approved by the Ethics Committee of Daping Hospital, Army Medical University (Chongqing, China), and adhered to the tenets of the Declaration of Helsinki. Orbital connective tissue samples were obtained as surgical waste during orbital decompression for acute TAO patients or blepharoplasty for healthy subjects. Peripheral venous blood samples were collected at the beginning of both TAO patients’ and healthy subjects’ admission.

### PD-L1 Treatment

Activated T cells were treated with either PBS, recombinant human PD-L1 protein (10 ug/mL; R&D Systems, Minneapolis, MN, USA), PD-L1 combined with goat anti-human IgG (10 ug/mL; R&D Systems, Minneapolis, MN, USA), or PD-L1 combined with PD-L1 neutralizing antibody (10 ug/mL; Abcam, Cambridge, UK), and the supernatants were subjected to ELISA after a 72-hour culture to detect the secretion of IFN-γ, IL-1β, TNF-α, and IL-2. Lymphocytes isolated from peripheral venous blood of TAO patients were treated with either PBS, recombinant human PD-L1 protein (10 ug/mL), PD-L1 combined with goat anti-human IgG (10 ug/mL), or PD-L1 combined with PD-L1 neutralizing antibody (10 ug/mL) for 48 hours, and the proportion of CD3+CD40L+ cells were determined by FCM. OFs from patients with TAO (TAO-OFs) were treated with either PBS, PD-L1 (10 ug/mL), autologous activated T cells (OFs: T cells = 1: 10), or PD-L1 combined with T cells for 24 hours. TAO-OFs were subjected to flow cytometry (FCM) and immunofluorescence staining (IF) for CD40 expression, and the total protein extracted from TAO-OF layers were subjected to WB to detect the expression levels of p38, ERK1/2, JNK, and NF-κB in TAO-OFs. After a 48-hour co-culturing, the supernatants were subjected to analysis of sICAM-1, IL-6, IL-8, and HA production by ELISA.

### Assessment of Cytokines and Hyaluronan Levels

Cytokines and Hyaluronan (HA) were assessed in triplicate by ELISA. TAO-OFs were treated with either PBS, IFN-γ (100 U/mL; Sino Biological Inc., Beijing, China), sCD40L (100 ng/mL; Sino Biological Inc., Beijing, China), or IFN-γ combined with sCD40L for 48 hours. TAO-OFs and CD40-knockdown TAO-OFs were co-cultured with autologous T cells (OFs: T cells = 1: 10) for 48 hours or not. TAO-OFs were stimulated with either PBS, IFN-γ neutralizing antibody (1 ug/mL; Proteintech, ORD, USA), autologous T cells, and IFN-γ Ab combined with and T cells for 48 hours. TAO-OFs were treated with either SB203580 (30 uM, p38 inhibitor), PD98059 (30 uM, ERK1/2 inhibitor), SP600125 (30 uM, JNK inhibitor), or PDTC (100 uM, NF-κB inhibitor) (all from MedChemExpress, NJ, USA) for 30 minutes, and then co-cultured with autologous T cells for 24 hours or not. Supernatant of cell culture from each experiment was collected and centrifuged at 1000 g for 10 minutes to remove debris. HA, IL-6, IL-8 (all from R&D Systems, Minneapolis, MN, USA), and sICAM-1 (4A Biotech, Beijing, China) were quantified in triplicate by ELISA according to the manufacturer’s protocols.

### Small Interfering RNA Transfection

TAO-OFs were plated and transiently transfected using Lipofectamine 3000 (Invitrogen, Carlsbad, CA, USA) with either 50 nM of CD40 siRNA or negative control siRNA (GeneBio Co., Shanghai, China) according to the manufacturer’s protocol. To detect the transfection efficiency, cells were incubated for 48 hours before being analyzed by RT-qPCR and incubated for 72 hours before being analyzed by FCM. TAO-OFs and CD40-knockdown TAO-OFs were co-cultured with T cells (OFs: T cells = 1: 10) for 24 hours or not, the total protein extracted from TAO-OF layers were subjected to western blot (WB) to detect the expression levels of p38, ERK1/2, JNK, and NF-κB in TAO-OFs.

### Analysis of CD40 Expression

The CD40 expression in TAO-OFs was detected by FCM and IF. TAO-OFs were treated with either PBS, IFN-γ (100 U/mL), IFN-γ (100 U/mL) combined with IFN-γ Ab (1 ug/mL), autologous T cells (OFs: T cells = 1: 10), IFN-γ Ab combined with T cells, or PD-L1 combined with T cells for 24 hours. Then, TAO-OFs were subjected to FCM and IF for CD40 expression.

### Statistics

Each experiment was repeated at least three times and data were graphed using GraphPad Prism Version 8.0 software (GraphPad Software, La Jolla, CA, USA). Statistical analyses were performed using SPSS version 23.0 software (IBM Corp., Armonk, NY, USA). Paired student’s *t*-test was used as appropriate. All values were expressed as mean ± SD and statistical significance was defined as values of *P* < 0.05 (*); *P* < 0.01 (**); and *P* < 0.001 (***).

## Results

### PD-L1 Inhibited the Activity of T cells

**Figure 1:**
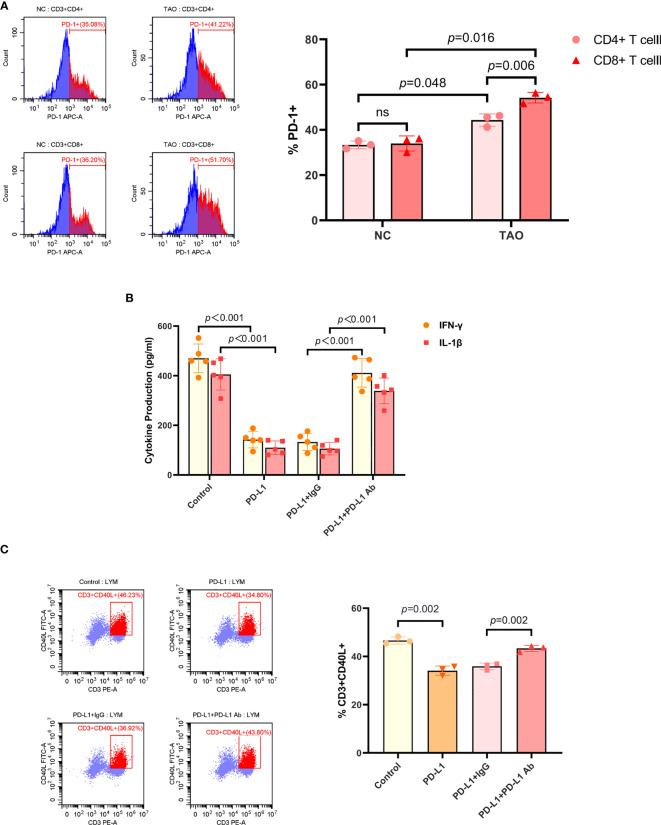
PD-L1 inhibited the activity of T cell. **(A)** Lymphocytes isolated from peripheral venous blood of TAO patients compared to negative controls were subjected to FCM to detect the proportion of PD-1+ cells in CD4+ T cells and CD8+ T cells. **(B)** T cells were treated with PBS, recombinant human PD-L1 protein (10 ug/mL), PD-L1 combined with goat anti-human IgG (10 ug/mL), and PD-L1 combined with PD-L1 neutralizing antibody (10 ug/mL), respectively. And the supernatants were subjected to ELISA after a 72-hour culture to detect the secretion of IFN-γ and IL-1β. **(C)** Lymphocytes isolated from peripheral venous blood of TAO patients were treated with PBS, recombinant human PD-L1 protein (10 ug/mL), PD-L1 combined with goat anti-human IgG (10 ug/mL), and PD-L1 combined with PD-L1 neutralizing antibody (10 ug/mL) for 48 hours, respectively. And the proportion of CD3+CD40L+ cells were determined by FCM. Data are expressed as mean ± SD of three or more repetitions. ns, no significance. Representative of three or more independent experiments using cells from a different donor.

As activated T cells have been proposed to express abundant PD-1 (13, 14), we initially determined the proportion of PD-1+ cells in CD4+ T cells and CD8+ T cells by FCM, respectively. Our results showed that the expression of PD-1 by CD4+ T cells and CD8+ T cells from peripheral venous blood of TAO patients was significantly higher than that of healthy subjects, and the proportion of PD-1+ cells in CD8+ T cells was higher than that in CD4+ T cells (**Figure 1A**).

The general model for the immunosuppression mediated by PD-1/PD-L1 is based on the interaction between PD-L1 on the tumor cells and PD-1 on T cells. It has been reported that PD-L1 treatment resulted in inhibiting T cell proliferation and decreased IL-2, IL-4, IL-10, IFN-γ, and TNF-α secretion (26). Therefore, we next sought to detect the inhibition of PD-L1 exerting on T cells. Recombinant human PD-L1 protein was used to treat T cells, and the supernatants were subjected to ELISA after a 48-hour culture. T cells included in lymphocytes, which were isolated from peripheral venous blood of TAO patients, were enriched and activated by anti-human CD3/CD28 monoclonal antibody beads (**Figures S1A, S1B**). It was found that the production of IFN-γ, IL-1β, TNF-α, and IL-2 in T cells significantly decreased after PD-L1 treatment, and the neutralizing antibody to PD-L1 inhibited this effect (**Figures 1B**, **S1C**). We then detected the expression of CD40L in T cells by FCM. The results showed that PD-L1 significantly inhibited the expression of CD40L in T cells, and the neutralizing antibody to PD-L1 blocked the inhibition too (**Figure 1C**).

### The Expression of PD-L1 in OFs and the Effect Exogenous PD-L1 Exerted on the OF-T Cell Co-Culture System

**Figure 2:**
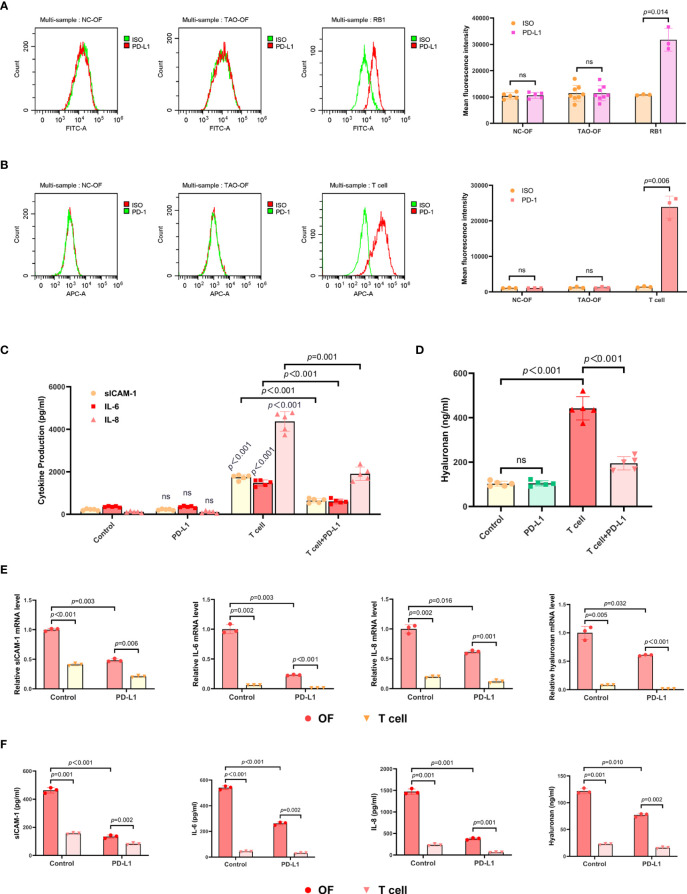
The expression of PD-L1 in OFs and the effect exogenous PD-L1 exerted on the OF-T cell co-culture system. **(A)** OFs from TAO patients and negative controls were subjected to FCM to detect the expression of PD-L1. **(B)** OFs from TAO patients and negative controls were subjected to FCM to detect the expression of PD-1. **(C, D)** TAO-OFs were stimulated with PBS, PD-L1 (10 ug/mL), autologous T cells (OFs: T cells = 1: 10), and PD-L1 combined with T cells for 48 hours, respectively. And the supernatants were subjected to analysis of sICAM-1, IL-6, IL-8, **(C)** and HA **(D)** production by ELISA. **(E, F)** TAO-OFs were co-cultured with autologous T cells (OFs: T cells = 1: 10) for 48 hours, and the two cells were subjected to qPCR to assay mRNA expressions of sICAM-1, IL-6, IL-8, and HA **(E)**, or to ELISA to detect the production of sICAM-1, IL-6, IL-8, and HA in cytoplasm **(F)**. Data are expressed as mean ± SD of three or more repetitions. ns, no significance. Representative of three or more independent experiments using cells from a different donor.

To investigate whether OFs expressed PD-L1, eight OF samples from TAO patients (TAO-OFs) and five OF samples from negative controls (NC-OFs) were collected and FCM was performed. RB1, a tumor cell line of retinoblastoma, was selected as a positive control in this experiment. It was found that PD-L1 was low expressed by OFs (**Figure 2A**). In addition, we also detected the expression of PD-1 in OFs with a positive control of activated T cells, and our data showed that PD-1 was low expressed in OFs too (**Figure 2B**).

Furthermore, the OF-T cell (activated T cells) co-culture system was treated with recombinant human PD-L1 protein for 48 hours, and the supernatants were subjected to ELISA. As shown in **Figures 2C, D**, **S2C**, the expressions of sICAM-1, IL-6, IL-8, CCL2, and HA in the OF-T cell co-culture system treated with PD-L1 were significantly decreased.

To figure out which cell (OF or T cell) secreted these cytokines and hyaluronan, we further examined these molecules in cytoplasm and the relevant mRNA. The results showed that T cells expressed a small amount of sICAM-1 which was much less than that of OFs, and hardly expressed IL-6, IL-8, and HA. The expressions of these molecules by OFs in OF-T cell co-culture system were significantly reduced upon PD-L1 treatment (**Figures 2E, F**).

### T Cells Induced the Production of sICAM-1, IL-6, IL-8, and HA in TAO-OFs *via* the CD40-CD40L Pathway

T cells induced the activation of TAO-OFs *via* the CD40-CD40L pathway, leading to considerably high expression of HA and cytokines (27–30). Therefore, we first examined the expression of CD40 in OFs. Five TAO-OF samples and five NC-OF samples were collected respectively and subjected to FCM. The results showed that NC-OFs expressed a small amount of CD40, and the expression of CD40 in TAO-OFs was significantly higher (**Figure S3A**).

**Figure 3:**
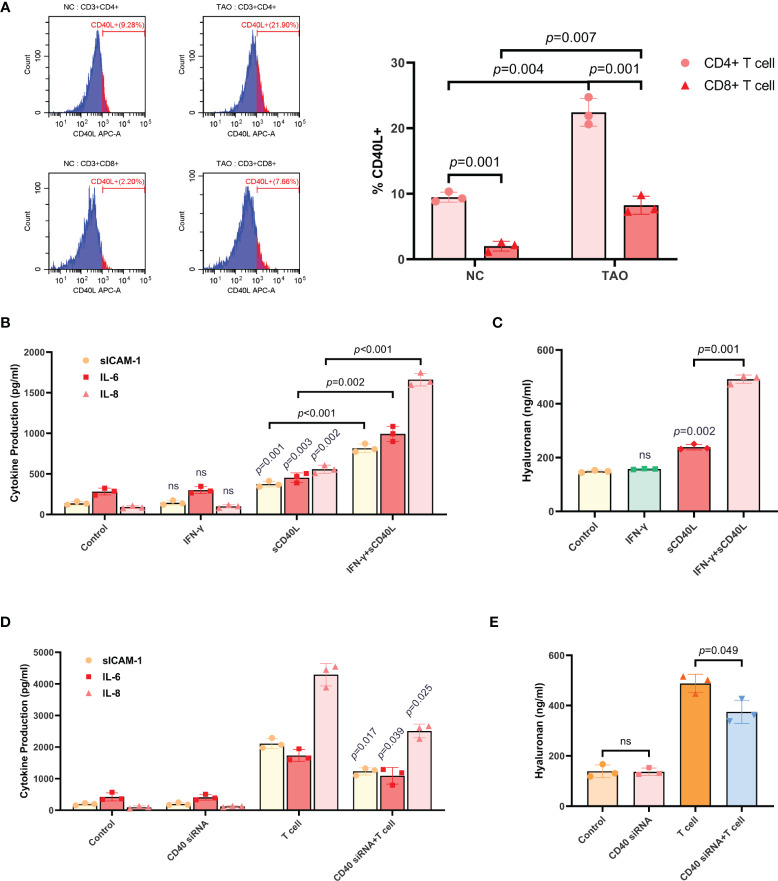
T cells induced the production of sICAM-1, IL-6, IL-8, and HA in TAO-OFs *via* the CD40-CD40L pathway. **(A)** FCM of lymphocytes isolated from peripheral venous blood of TAO patients compared to negative controls demonstrating differential proportion of CD40L+ cells in CD4+ T cells and CD8+ T cells. **(B, C)** TAO-OFs were treated with PBS, IFN-γ (100 U/mL), sCD40L (100 ng/mL), and IFN-γ combined with sCD40L for 48 hours, respectively. And the supernatants were subjected to analysis of sICAM-1, IL-6, IL-8, **(B)** and HA **(C)** contents by ELISA. **(D, E)** TAO-OFs and CD40-knockdown TAO-OFs were co-cultured with autologous T cells (OFs: T cells = 1: 10) for 48 hours or not, and the supernatants were subjected to ELISA to detect the production of sICAM-1, IL-6, IL-8, **(D)** and HA **(E)**. Data are expressed as mean ± SD of triplicates. ns: no significance. Representative of three independent experiments using cells from a different donor.

Peripheral venous blood samples from TAO patients and negative controls were collected, then lymphocytes were isolated and subjected to FCM. The results showed that CD4+ T cells from negative controls expressed a small amount of CD40L, whereas CD8+ T cells almost did not, and the proportion of CD40L+ cells in either CD4+ T cells or CD8+ T cells of TAO samples was increased apparently (**Figure 3A**).

As depicted above, CD40 was involved in stimulating HA and cytokine synthesis (11, 27–30). To verify this conclusion, we first treated TAO-OFs with either sCD40L, IFN-γ, or the combination. The supernatants were subjected to analysis of HA and cytokines contents by ELISA. It was found that, sCD40L, but not IFN-γ, slightly increased the secretion of sICAM-1, IL-6, IL-8, and HA. While significant increases of these cytokines and HA could be detected in TAO-OFs treated with sCD40L combined with IFN-γ (**Figures 3B, C**).

Then, CD40-knockdown TAO-OFs were used in the experiments. CD40 siRNA was transiently transfected into TAO-OFs. Cell monolayers were collected for total RNA extraction after a 48-hour culture, and RT- qPCR was performed. As **Figure S3B** showed, TAO-OFs transfected with CD40 siRNA displayed decreased levels of CD40 mRNA under autologous T cell stimulation compared with TAO-OFs transfected with NC siRNA. In addition, cells cultured for 72 hours were subjected to FCM and the results revealed that the expression of CD40 protein in TAO-OFs transfected with CD40 siRNA was also significantly reduced (**Figure S3C**). We co-cultured autologous T cells with CD40 knock-down TAO-OFs, and the production of cytokines and HA were assessed by ELISA. As shown in **Figures 3D, E**, no significant difference was detected in the production of cytokines or HA between the CD40 siRNA transfected group and the negative control group. While they were significantly increased when stimulated by T cells. However, they showed relatively lower increases in the group transfected with CD40 siRNA followed by T cell stimulation.

### T Cells Up-Regulated the Expression of CD40 in TAO-OFs by Secreting IFN-γ

**Figure 4:**
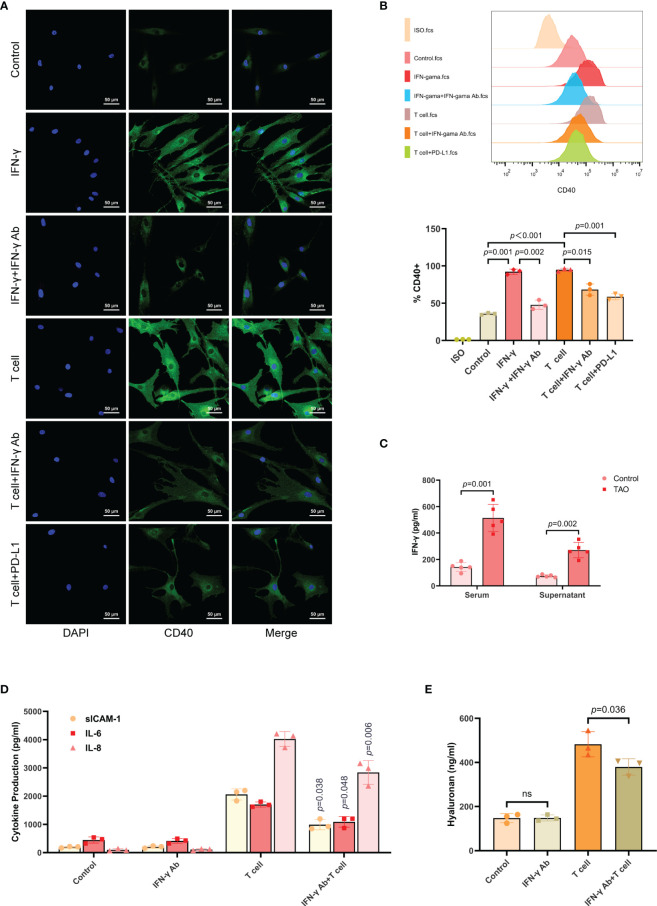
T cells up-regulated the expression of CD40 in TAO-OFs by secreting IFN-γ. **(A, B)** TAO-OFs were stimulated with PBS, IFN-γ (100 U/mL), IFN-γ combined with IFN-γ neutralizing antibody (1 ug/mL), autologous T cells (OFs: T cells = 1: 10), T cells combined with IFN-γ Ab, and T cells combined with PD-L1 for 24 hours, respectively. And TAO-OFs were subjected to IF **(A)** and FCM **(B)** for CD40 expression. **(C)** Comparison of IFN-γ concentration in serum and lymphocyte culture supernatants between TAO patients and negative controls by ELISA. Lymphocytes were cultured in RPMI 1640 containing 10% FBS for 72 hours. **(D, E)** TAO-OFs were stimulated with PBS, IFN-γ Ab (1 ug/mL), autologous T cells, and T cells combined with IFN-γ Ab for 48 hours, respectively. And the supernatants were subjected to ELISA for sICAM-1, IL-6, IL-8, **(E)** and HA **(D)** production. Data are expressed as mean ± SD of three or more repetitions. ns, no significance. Representative of three or more independent experiments using cells from a different donor.

Activated T cells were known to secrete IFN-γ (31, 32). Additionally, it has been reported that IFN-γ stimulated the overexpression of CD40 in TAO-OFs (27–29). To verify this view, we first stimulated TAO-OFs with autologous T cells (OFs: T cells = 1: 10), T cells combined with IFN-γ neutralizing antibody, or T cells combined with recombinant human PD-L1 protein, and then detected the expression of CD40 in TAO-OFs by IF and FCM. IFN-γ-treated TAO-OFs and untreated TAO-OFs were used as the positive control and the negative control, respectively. It was shown that the expression of CD40 in TAO-OFs increased significantly when stimulated by autologous T cells, while both IFN-γ Ab and PD-L1 inhibited the stimulation (**Figures 4A, B**).

Then, we detected serum IFN-γ levels from TAO patients and negative controls by ELISA. It was found that the content of IFN-γ in serum from TAO patients was significantly high than that of negative controls. The IFN-γ content of PBMCs culture supernatant was also measured and the analogous result was obtained (**Figure 4C**).

As showed above, T cells induced the production of cytokines and HA in TAO-OFs *via* the CD40-CD40L pathway. Hence, we utilized IFN-γ neutralizing antibody to treat the OF-T cell co-culture system, and the results showed the production of sICAM-1, IL-6, IL-8, and HA in TAO-OFs stimulated by T cells after blocking IFN-γ were also reduced (**Figures 4D, E**).

### PD-L1 Down-Regulated the Phosphorylation Levels of MAPK and NF-κB Pathway Proteins in TAO-OFs From the OF-T Cell Co-Culture System *via* the CD40-CD40L Pathway

**Figure 5:**
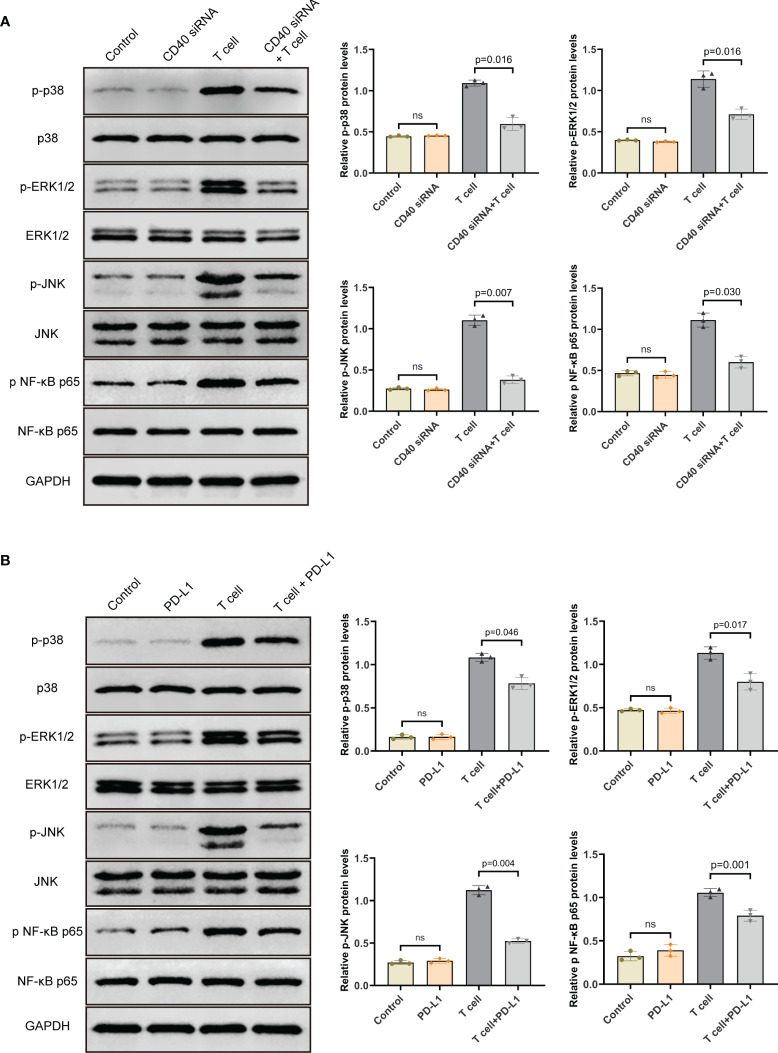
PD-L1 down-regulated the phosphorylation levels of MAPK and NF-κB pathway proteins in TAO-OFs from the OF-T cell co-culture system *via* the CD40-CD40L pathway. **(A)** TAO-OFs and CD40-knockdown TAO-OFs were co-cultured with autologous T cells (OFs: T cells = 1: 10) for 24 hours or not, the total protein extracted from TAO-OF layers was subjected to western blot to detect the expression levels of p38, ERK1/2, JNK and NF-κB p65 in TAO-OFs. **(B)** TAO-OFs were treated with PBS, PD-L1 (10 ug/mL), autologous T cells (OFs: T cells = 1: 10), and T cells combined with PD-L1 for 24 hours, respectively. And the total protein extracted from TAO-OF layers was subjected to western blot to detect the expression levels of p38, ERK1/2, JNK and NF-κB p65 in TAO-OFs. Data are expressed as mean ± SD of triplicates. ns, no significance. Representative of three independent experiments using cells from a different donor.

To investigate how PD-L1 affected the pathways in TAO-OFs from the OF-T cell co-culture system, we first analyzed the effect of CD40 on the expression of the phosphorylation of MAPK and NF-κB signaling proteins in TAO-OFs. After transfection of CD40 siRNA followed by a 24-hour co-culture with autologous T cells, the TAO-OF layers were harvested for total protein extraction and the expression levels of MAPKs (p38, ERK1/2, JNK) and NF-κB p65 were detected by western blot. It was found that there was no significant difference in the expressions of these signaling proteins in the group transfected with CD40 siRNA compared with the negative control group. However, the phosphorylation levels of all the signaling proteins were significantly increased when stimulated by T cells. In contrast, TAO-OFs transfected with CD40 siRNA under the stimulation of T cells showed relatively lower phosphorylation levels of the four signaling proteins (**Figure 5A**).

In the second place, the OF-T cell co-culture system was treated with PD-L1 for 24 hours, and the total protein extracted from TAO-OF layers was subjected to western blot. Our data showed that PD-L1 had no direct effect on the expression of total p38, ERK1/2, JNK and NF-κB p65 proteins. However, compared with the untreated group, the phosphorylation levels of these signaling proteins in TAO-OFs from the OF-T cell co-culture system treated with PD-L1 were significantly down-regulated (**Figure 5B**).

### The Production of sICAM-1, IL-6, IL-8, and HA in TAO-OFs From the OF-T Cell Co-Culture System Were Regulated by the MAPK and NF-κB Signaling Pathways

**Figure 6:**
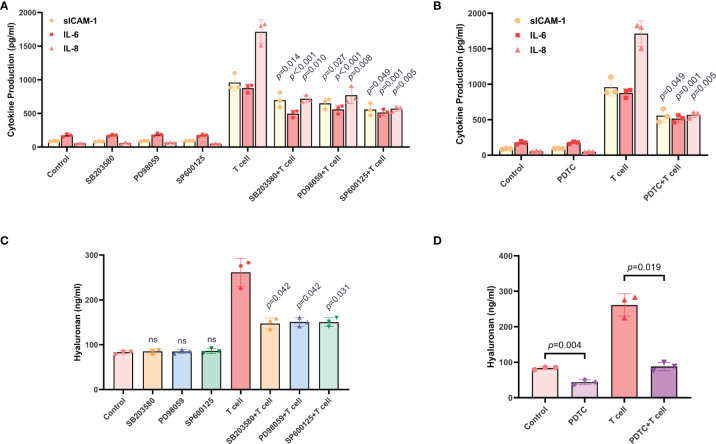
The production of sICAM-1, IL-6, IL-8, and HA in TAO-OFs from the OF-T cell co-culture system were regulated by the MAPK and NF-κB signaling pathways. **(A–D)** TAO-OFs were treated with SB203580 (30 uM), PD98059 (30 uM), SP600125 (30 uM), and PDTC (100 uM) for 30 minutes, respectively, and then co-cultured with autologous T cells (OFs: T cells = 1: 10) for 24 hours or not. The supernatants were subjected to analysis of sICAM-1, IL-6, IL-8, **(A, B)** and HA **(C, D)** production by ELISA. Data are expressed as mean ± SD of triplicates. ns, no significance. Representative of three independent experiments using cells from a different donor.

Based on several previous reports demonstrating the involvements of the MAPK and NF-κB signaling pathways in the production of sICAM-1, IL-6 and IL-8 by TAO-OFs (27, 33–36), we further investigated the role of MAPK and NF-κB signaling pathways in T cell-induced cytokines secretion in TAO-OFs. TAO-OFs were treated with either SB203580, PD98059, SP600125, or PDTC for 30 minutes, and then co-cultured with autologous T cells for 24 hours or not. The supernatants were subjected to ELISA subsequently. The data demonstrated that the production of sICAM-1, IL-6, IL-8, and CCL2 did not change in the supernatants from TAO-OFs treated with inhibitors only, whereas, they were significantly reduced in the supernatants from TAO-OFs treated with inhibitors followed by co-culturing with T cells (**Figures 6A, B**, **S2D**). Furthermore, the production of HA in co-culture groups treated with inhibitors was significantly decreased (**Figures 6C, D**). In addition, no correlation could be detected between HA secretion and MAPK inhibitors without T cell stimulation (**Figure 6C**), however, the secretion of HA was inhibited by PDTC alone (**Figure 6D**).

## Discussion

**Figure 7:**
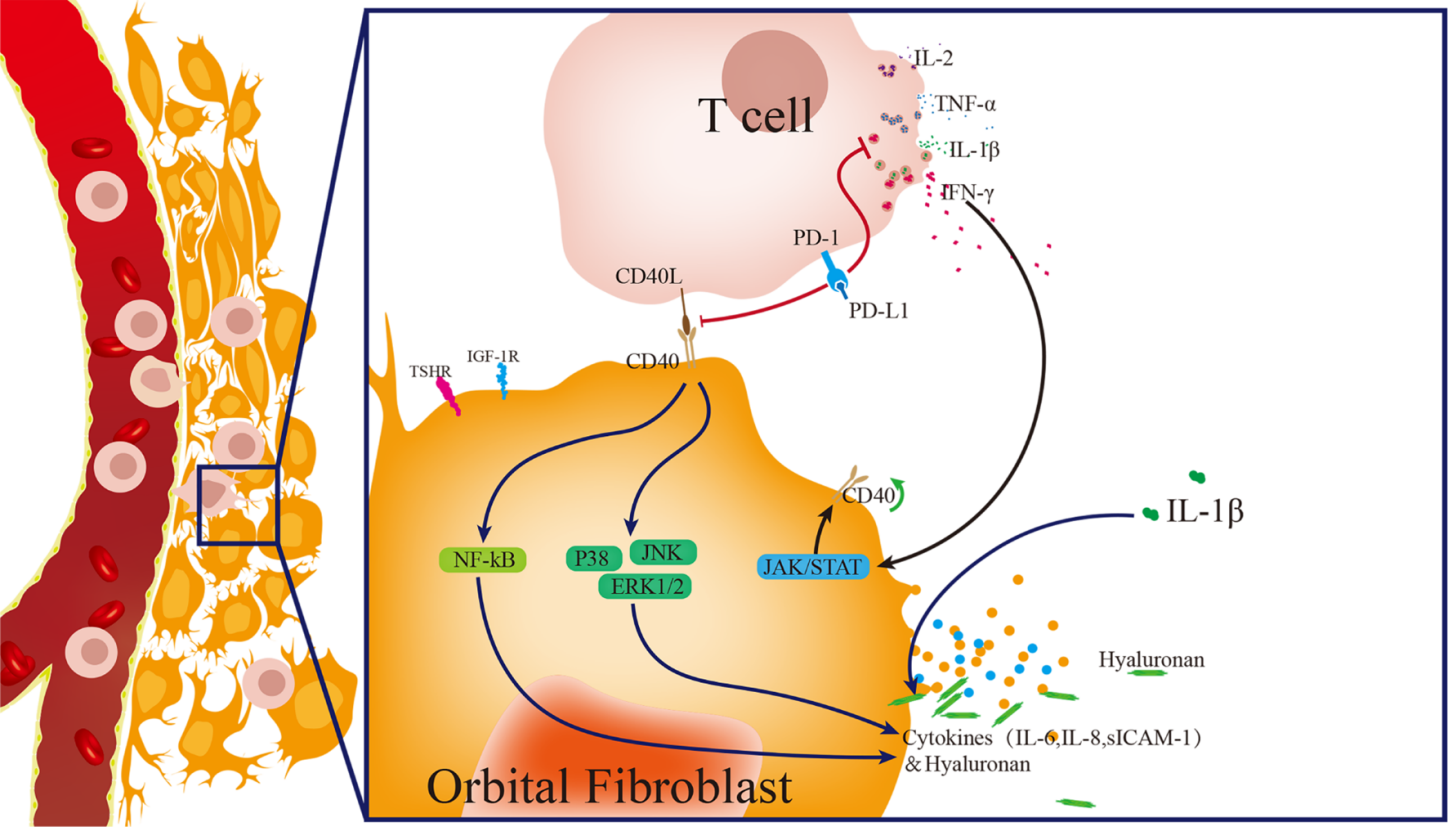
Model of pathogenesis of TAO induced by T cells activating OFs, and PD-L1 blocking the progression of TAO. Activated T cells express abundant CD40L and release a large amount of cytokines, such as IL-1β, IL-2, IFN-γ and TNF-α. IFN-γ up-regulates the expression of CD40 in OFs. IL-1β induces the high expression of cytokines in OFs. T cells activate OFs *via* CD40-CD40L costimulatory molecules, thus up-regulating the phosphorylation of the downstream pathway proteins, including p38, ERK 1/2, JNK and NF-κB p65, and inducing the high production of sICAM-1, IL-6, IL-8, and hyaluronan. PD-L1 inhibits T cell activity, and thus weakens T cell-induced OFs activation, thereby significantly reducing the production of cytokines and hyaluronan in OFs.

PD-1/PD-L1, a crucial inhibitory signaling pathway in regulating T cell activity, functions as a suppressor in the progression of Graves’ disease. However, the role of the PD-1/PD-L1 pathway in the pathogenesis of TAO remains unclear. In the present study, we determine that TAO-OFs do not express PD-L1, and exogenous PD-L1 attenuates CD40-CD40L-mediated T cells activating TAO-OFs by inhibiting T cell function, thus inhibiting immune inflammation and HA aggregation in the orbit of TAO patients (**Figure 7**).

T cells are the key immune cells that induce the activation of OFs and promote the development of TAO (11). In the clinic, the use of some broad-spectrum immunosuppressants, such as corticosteroids, in the treatment of TAO can significantly inhibit T cell activity (4, 5). But if the activity of T cells can be inhibited in a targeted and safe way, we might achieve a better therapeutic result. Turning attention to tumor immunity, we focus on PD-1/PD-L1, a negative immune costimulatory pathway, which plays a vital role in protecting tumor cells from immune attack (13, 14). PD-L1 inhibits the activation of T cells and reduces the cytokines secretion of T cells (13, 14). Our data shows that PD-L1 also inhibits the expression of CD40L in T cells, which is in line with the finding in an inflammatory model (37). Recent studies suggested that PD-1/PD-L1 could partially inhibit the pathogenesis of GD (22–25). Moreover, the application of a PD-1 inhibitor resulted in some symptoms similar to TAO (21). We, therefore, wonder whether PD-1/PD-L1 also plays a certain inhibitory role in the pathogenesis of TAO. In the present study, it is found that PD-1 and PD-L1 were low expressed on the surface of OFs from both TAO patients and negative controls. This result suggests that the immune tolerance mechanism of the PD-1/PD-L1 pathway is absent in OFs, which may be one of the causes for the active orbital inflammatory response in TAO patients. Previous studies have demonstrated that CD34+ orbital fibroblasts (OFs), migrating from the circulation into the orbit of TAO patients but not healthy subjects, were the crucial cells that initiate the onset of TAO by expressing abundant cytokines and differentiating further into myofibroblasts and adipocytes (1, 3, 38). We then attempt to construct a co-culture system of TAO-OFs and activated T cells upon PD-L1 treatment. Our data indicate that exogenous PD-L1 significantly reduces the production of sICAM-1, IL-6, IL-8, CCL2, and HA in the OF-T cell co-culture system. We have conducted experiments to examine the levels of sICAM-1, IL-6, IL-8, and hyaluronan from NC-OFs co-cultured with activated T cells upon PD-L1 treatment. The results showed significant differences of these molecule expressions between NC-OFs versus TAO-OFs co-cultured with activated T cells either upon PD-L1 treatment or not. Additionally, the relative reduced expression of CD40 and phosphorylation levels of MAPKs and NF-κB p65 are also observed in TAO-OFs.

Previous reports suggested that activated T cells induce the proliferation of TAO-OFs (39), and significant increases of sICAM-1, IL-6 and IL-8 could be induced by sCD40L combined with IFN-γ in TAO-OFs (11, 28–30). In this study, the production of HA is also up-regulated. Our results also indicate that the secretions of these cytokines and HA in TAO-OFs are significantly up-regulated by activated T cells (40–42). It has been reported that CD40 was highly expressed in TAO-OFs compared with NC-OFs, and IFN-γ stimulated the overexpression of CD40 in TAO-OFs (27–29). T cells also induce the increased CD40 expression of TAO-OFs in our study. Therefore, we speculate that the high expression of CD40 in TAO-OFs may be due to the active immune system, where activated T cells infiltrate into the orbit and secrete large amounts of IFN-γ to up-regulate the expression of CD40 in TAO-OFs. In addition, the proportion of CD40L+ cells was increased in lymphocytes from TAO patients. It’s clear that CD40L, highly expressed on the surface of T cells, binding to CD40 expressed on the surface of TAO-OFs, stimulates the activation of TAO-OFs and induces the production of cytokines and HA.

CD40-CD40L, also known as a pair of costimulatory molecules, is essential for cellular immune responses (43). It has also been shown to act as an upstream master switch for the MAPK and NF-κB signaling pathways (43–45). Previous studies showed that sCD40L stimulated the secretion of cytokines and HA by up-regulating the phosphorylation levels of MAPK and NF-κB signaling pathways in TAO-OFs (27, 29, 33–36, 46). Our results indicate that activated T cells show analogous effects. When the CD40 gene of TAO-OFs was knocked down, the phosphorylation levels of p38, ERK1/2, JNK, and NF-κB p65 induced by T cells were significantly decreased, as well as the production of cytokines and HA. In addition, T cell-induced production of cytokines and HA in TAO-OFs were also reduced by inhibitors of MAPK and NF-κB p65 pathway proteins. These results suggest that T cells up-regulate the phosphorylation levels of MAPK and NF-κB signaling pathways in TAO-OFs *via* the CD40-CD40L costimulatory pathway, thus inducing the production of cytokines and HA.

Previous studies reported that MAPKs activation led to a substantial increase of HA synthesis in TAO-OFs (29, 41, 47). However, a contrary conclusion was presented in another research (48), which suggested PD98059, an ERK1/2 inhibitor, up-regulating IGF-1-induced HA secretion in TAO-OFs. Due to the shortage of researches in this aspect, the causes for this divergence are unclear, and the distinction of stimuli may be taken into account. Furthermore, our results show that the secretion of HA in TAO-OFs is also closely associated with the NF-κB pathway, which has never been reported in TAO before. The conclusions drawn by several studies in other models (49–51), including aortic smooth muscle cells, endothelial cells and type-B synoviocytes, were in agreement with it. Interestingly, our data suggest that PDTC not only inhibits T cell-induced HA secretion in TAO-OFs but also decreases the basal HA secretion of TAO-OFs. This is not consistent with a previous study with endothelial cells (51). One reason for this may be that TAO-OFs can secrete a small number of cytokines, including IL-1β, without external stimulus, which in turn induce the production of HA in TAO-OFs *via* activating the NF-κB pathway, while the basal secretion of HA in endothelial cells is relatively lower.

These results suggest that PD-L1 can block T cell-induced activation of TAO-OFs in two ways. PD-L1 not only down-regulates CD40L expression in T cells but also reduces IFN-γ secretion, thus attenuating T cells stimulated CD40 expression in TAO-OFs. In other words, PD-L1 inhibits the activity of T cells, and thus weakens T cell-induced TAO-OF activation *via* the CD40-CD40L costimulatory pathway, thereby down-regulating the phosphorylation levels of p38, ERK1/2, JNK, and NF-κB p65 pathway proteins, and significantly reducing the production of sICAM-1, IL-6, IL-8, and HA in TAO-OFs (**Figure 7**). Therefore, the reconstruction of immune tolerance utilizing the PD-1/PD-L1 pathway may appear to be a novel approach for the treatment of TAO and other autoimmune diseases in the future.

In this study, the pathological mechanism of T cell-activated OFs initiating the pathogenesis of TAO has been revealed preliminarily. However, the role of TSHR and IGF-1R, the common antigens of TAO, in the pathogenesis of TAO have not been discussed. Additionally, we have shown that PD-L1 can be applied to efficiently inhibit T cell-induced production of cytokines and HA in TAO-OFs *in vitro*. However, considering the current TAO animal modeling methods are inefficient and difficult to replicate (52–54), further verification has not been performed *in vivo* for a firm conclusion.

In summary, the current study presents persuasive evidence that the PD-1/PD-L1 pathway is deficient in the orbit of both TAO patients and healthy subjects, and explores the possibility of utilizing exogenous PD-L1 to reconstruct the orbital immune tolerance microenvironment in patients with TAO. More importantly, the application of PD-L1 *in vitro* weakens T cell-induced OF activation by inhibiting T cell activity, thus inhibiting immune-inflammatory reaction and HA aggregation in the orbital region of TAO patients. These findings may provide new intervention strategies for the pathogenesis of TAO, and may also be expected to shed light on the treatment of other autoimmune diseases.

## Data Availability Statement

The raw data supporting the conclusions of this article will be made available by the authors, without undue reservation.

## Ethics Statement

The studies involving human participants were reviewed and approved by the Ethics Committee of Daping Hospital, Army Medical University, Chongqing, China. The patients/participants provided their written informed consent to participate in this study.

## Author Contributions

ZBL and YL contributed equally to this paper. ZBL, XZ, and HFY formulated the concept and designed the study. XB, XYG, and HFY collected the specimen. ZBL, YL, QJG, and BH performed the experiments. MML, AJS, and XHL analyzed the data. ZBL and YL drafted the paper. ZBL, YL, FLL, XZ and HFY participated in discussions associated with the paper. XZ and HFY revised the paper. All authors read and approved the submitted manuscript.

## Funding

Supported by the Chongqing Science and Technology Program (Grant No. 30119-2961), the Talent Innovation Capacity Development Program of Army Medical Center (Grant No. 5012002-3519) and the Science and Technology Research Program of Chongqing Municipal Education Commission (Grant No. KJQN201901138).

## Conflict of Interest

The authors declare that the research was conducted in the absence of any commercial or financial relationships that could be construed as a potential conflict of interest.

## Publisher’s Note

All claims expressed in this article are solely those of the authors and do not necessarily represent those of their affiliated organizations, or those of the publisher, the editors and the reviewers. Any product that may be evaluated in this article, or claim that may be made by its manufacturer, is not guaranteed or endorsed by the publisher.
