# Supplementary material for: PD-L1 Inhibits T Cell-Induced Cytokines and Hyaluronan Expression via the CD40-CD40L Pathway in Orbital Fibroblasts From Patients With Thyroid Associated Ophthalmopathy
Source: Front Immunol. 2022 May 10;13:849480. doi: 10.3389/fimmu.2022.849480 (PMC9128409; doi:10.3389/fimmu.2022.849480)
Supplement: Supplementary file 1 [file DataSheet_1.docx]

**SUPPLEMENTARY MATERIALS and METHODS**

**Fibroblasts and T cells Culture**

Primary human OFs were cultured from orbital connective tissue. Tissue explants attached to plastic culture dishes and were covered with Dulbecco’s modified Eagle’s medium (DMEM; Corning, NY, USA) containing 20% fetal bovine serum (FBS; PAN, Adenbach, Germany) and 5% Penicillin-Streptomycin Solution. They were incubated in a humidified 37°C incubator with 5% CO_2_ environment. Once a fibroblast monolayer was obtained, they were serially passaged with gentle trypsin/EDTA treatment. Fibroblasts after the second passage were cultured in DMEM medium with 10% FBS. Medium was changed every 2 to 4 days, and cells were used between the 2nd and 5th passages from culture initiation.

Lymphocytes were isolated from 10 mL of peripheral venous blood by gradient centrifugation. T cells were enriched and activated from lymphocytes using anti-human CD3/CD28 monoclonal antibody beads (T&L Biological Technology CO., Beijing, China) according to the manufacturer’s instructions. The activated T cells were expanded by co-culturing for 8 days with CD3/CD28 beads in RPMI 1640 (Corning, NY, USA) containing 10% FBS and supplemented with recombinant human IL-2 (100 U/mL; Sino Biological Inc., Beijing, China) on days 0 and 4. Beads were eluted on day 9-12 and the expanded T cells were readied for subsequent experiments.

**Flow Cytometry (FCM)**

Cells were harvested and placed in 1.5ml-eppendorf tubes, and cell suspensions were adjusted to a concentration of 10^6^ cells/ml in ice cold PBS, 10% FCS, 1% sodium azide. OFs were incubated with rabbit IgG, isotype control (Abcam, Cambridge, UK) or rabbit anti-human CD40 monoclonal antibodies (Abcam, Cambridge, UK) for 45minutes at 4 ℃ followed by a 3-time wash with ice cold PBS by centrifugation at 400 g for 5 minutes. Then cells were stained with AF488-conjugated goat anti-rabbit IgG (Abcam, Cambridge, UK) in the dark for 25 minutes at 4 ℃ and washed 3 times again. Cells were resuspended in 500 µl ice cold PBS and kept in the dark on ice or at 4 °C in a fridge until analysis. Prepared T cells were collected and stained with PE-conjugated CD3 monoclonal antibodies (OKT3) (eBioscience, San Diego, CA, USA) and APC-conjugated CD40L monoclonal antibodies (eBioscience, San Diego, CA, USA) for 30minutes at 4 ℃ in the dark. Cells were resuspended in 500 µl ice cold PBS following a 3-time wash. Analysis was performed within 24 hours using a flow cytometer (CytoFLEX; Beckman Coulter Inc, CA USA).

**Enzyme-Linked Immunosorbent Assays (ELISA)**

HA, IFN-γ, IL-1β, TNF-α, IL-2, IL-6, IL-8 (all from R&D Systems, Minneapolis, MN, USA), sICAM-1 (4A Biotech, Beijing, China) were quantified in triplicate by ELISA. Supernatants of cell culture were collected and centrifuged at 1000g for 10 minutes to remove debris. Remove excess microplate strips from the plate frame, return them to the foil pouch containing the desiccant pack, and reseal. Add 50 μL of Assay Diluent to each well. Mix well before and during use. Add 50 μL of standard, control, or sample per well. Cover with the adhesive strip provided. Incubate for 2 hours at room temperature on a horizontal orbital microplate shaker (0.12" orbit) set at 500 ± 50 rpm. Aspirate each well and wash, repeating the process four times for a total of five washes. Wash by filling each well with Wash Buffer (400 μL) using a squirt bottle, manifold dispenser, or autowasher. After the last wash, remove any remaining Wash Buffer by aspirating or decanting. Invert the plate and blot it against clean paper towels. Add 100 μL of Conjugate solution to each well. Cover with a new adhesive strip. Incubate for 2 hours at room temperature on the shaker. Repeat the aspiration/wash as described above. Add 100 μL of Substrate Solution to each well. Incubate for 30 minutes at room temperature on the benchtop (Protect from light). Add 100 μL of Stop Solution to each well. Gently tap the plate to ensure thorough mixing. Determine the optical density of each well within 30 minutes, using a microplate reader set to 450 nm. If wavelength correction is available, set to 540 nm or 570 nm. If wavelength correction is not available, subtract readings at 540 nm or 570 nm from the readings at 450 nm.

**Real-Time Quantitative PCR (RT-qPCR)**

Total RNA was extracted using the RNeasy Mini Kit (Qiagen; Dusseldorf, Germany), and reversely transcribed into cDNA with a commercial kit (SureScript™ First-Strand cDNA Synthesis Kit; GeneCopoeia, Guangzhou, China).

RT-qPCR was performed on a real-time PCR machine (Bio-Rad CFX96; Bio-Rad Laboratories, Inc., CA, USA) using a commercial kit (BlazeTaq™ SYBR® Green qPCR Mix 2.0; GeneCopoeia, Guangzhou, China) according to the manufacturer’s specifications. The primers used were as follows: CD40: forward, 5’- GGTTTCTGATACCATCTGCGA-3’ and reverse, 5’- CTTTGGTCTCACAGCTTGTCC-3’; GAPDH: forward, 5’-GGAGCGAGATCCCTCCAAAAT-3’ and reverse, 5’-GGCTGTTGTCATACTTCTCATGG-3’. The standard PCR conditions were 30 seconds at 95 ℃, followed by 40 cycles of 95 ℃ for 5 seconds, and 60 ℃ for 30 seconds. All reactions were carried out in triplicate. GAPDH was used as an internal control and the relative mRNA expression levels were analyzed using the 2^-ΔΔCt^ method.

**Immunofluorescence Staining (IF)**

TAO-OFs cultured on Millicell EZ SLIDE 8-well glass (Merck KGaA, Darmstadt, Germany) were fixed with 4% paraformaldehyde for 20 minutes, permeated with 0.3% Triton for 5 minutes, blocked with immunol staining blocking buffer (Beyotime, Shanghai, China) for 1 hour at room temperature, and then incubated overnight at 4 ℃ with rabbit anti-human CD40 monoclonal antibodies or rabbit IgG, isotype control. After three washes in PBS, the cells were incubated with AF488-conjugated goat anti-rabbit IgG for 1 hour at room temperature in the dark. Nuclei were stained for 5 minutes with antifade mounting medium with DAPI (Beyotime, Shanghai, China). A high resolution fluorescence microscope (IX83; Olympus Corporation, Tokyo, Japan) was used for imaging.

**Western Blot Analysis (WB)**

TAO-OFs were harvested and lysed on ice for 30 minutes in cell lysis buffer containing protease inhibitor, phosphatase inhibitor and PMSF. Cell lysates were then centrifuged at 12,000g and 4 ℃ for 15 min. The total protein concentrations were normalized with a bicinchoninic acid (BCA) kit (Solarbio, Beijing, China). Proteins were separated by SDS-PAGE and transferred onto PVDF membranes. The membranes were blocked with a commercial blocking buffer (QuickBlock™ Blocking Buffer for Western Blot; Beyotime, Shanghai, China) at room temperature for 15 minutes and then incubated at 4 ℃ overnight with primary rabbit anti-human total or phosphorylated p38, ERK1/2, JNK, NF-κB antibodies (all from Cell Signaling Technology, Boston, MA, USA) or GAPDH antibody (Sangon Biotech, Shanghai, China). After three washes with TBST, membranes were incubated with IRDye^®^ 800CW goat anti-rabbit IgG secondary antibody (LI-COR, Lincoln, Nebraska, USA) at room temperature for 60 minutes. The membranes were visually developed and photographed with a commercial imaging system (Odyssey Fc; LI-COR, Lincoln, Nebraska, USA), and the target protein bands were quantitatively analyzed using ImageJ software (Bethesda, MD, USA).

**SUPPLEMENTARY TABLES and LEGENDS**

**Table S1.** The activity assessement of TAO patients recruited in this study by the clinical activity score (CAS). CAS ≥ 3: active TAO; CAS < 3: inactive TAO.

| **No.** | **Gender** | **Age(Y)** | **Symptoms & signs** | | | | | | | **CAS** |
| --- | --- | --- | --- | --- | --- | --- | --- | --- | --- | --- |
|  |  |  | **SRP** | **PAG** | **RE** | **RC** | **SCP** | **SE** | **SC** |  |
| 1 | Female | 45 | 0 | 1 | 1 | 1 | 1 | 1 | 1 | 6 |
| 2 | Female | 52 | 0 | 0 | 0 | 1 | 0 | 1 | 1 | 3 |
| 3 | Male | 42 | 0 | 1 | 0 | 1 | 0 | 1 | 1 | 4 |
| 4 | Female | 46 | 1 | 1 | 1 | 1 | 1 | 0 | 1 | 6 |
| 5 | Male | 33 | 0 | 0 | 1 | 1 | 1 | 1 | 1 | 5 |
| 6 | Male | 56 | 0 | 1 | 1 | 1 | 0 | 0 | 0 | 3 |
| 7 | Female | 37 | 0 | 1 | 1 | 1 | 0 | 0 | 1 | 4 |
| 8 | Female | 57 | 0 | 1 | 0 | 1 | 1 | 0 | 1 | 4 |

Note. SRP: Spontaneous retrobulbar pain; PAG: Pain on attempted upward or downward gaze; RE: Redness of eyelids; RC: Redness of conjunctiva; SCP: Swelling of caruncle or plica; SE: Swelling of eyelids; SC: Swelling of conjunctiva.

**Table S2.** General information of the healthy subjects recruited in this study.

| **No.** | **Gender** | **Age(Y)** | **Diagnosis** | **Surgical method** |
| --- | --- | --- | --- | --- |
| 1 | Female | 62 | lower eyelid bag | Lower Eyelid Blepharoplasty |
| 2 | Female | 35 | prolapse of orbital fat | Resection of orbital fat prolapse |
| 3 | Male | 49 | lower eyelid bag | Lower Eyelid Blepharoplasty |
| 4 | Female | 56 | lower eyelid bag | Lower Eyelid Blepharoplasty |
| 5 | Male | 55 | lower eyelid bag | Lower Eyelid Blepharoplasty |

Note. All the subjects have no thyroid disorder or other autoimmune diseases.

**Table S3.** Specific information of the antibodies, recombinant proteins, and inhibitors used in this study.

| **No.** | **Category** | **Manufacturer** | **Technique** | **Concentration/Dilution** |
| --- | --- | --- | --- | --- |
| 1 | CD3 Ab-PE | Invitrogen | FCM | 5 µL (0.5 µg)/test |
| 2 | CD3 Ab-CL488 | Proteintech | FCM | 5 µL /test |
| 3 | CD4 Ab-CL488 | Proteintech | FCM | 1:100 |
| 4 | CD8 Ab-CL488 | Proteintech | FCM | 1:100 |
| 5 | PD-1 Ab-CL647 | Proteintech | FCM | 1:100 |
| 6 | goat anti-human IgG | R&D | ELISA | 10 ug/ml |
| 7 | PD-L1 Ab | Abcam | ALL | 10 ug/ml |
| 8 | CD40L Ab-APC | Invitrogen | FCM | 5 µL (0.5 µg)/test |
| 9 | IFN-γ Ab | Proteintech | ELISA&IF | 1 ug/mL |
| 10 | CD40 Ab | Abcam | IF&FCM | 1:100 & 1:500 |
| 11 | CD25 Ab-PE | Proteintech | FCM | 5 µL (0.06 µg)/test |
| 12 | rabbit IgG, isotype control | Abcam | FCM | 1:500 |
| 13 | goat anti-rabbit IgG-AF488 | Abcam | IF&FCM | 1:200 & 1:500 |
| 14 | p38 MAPK Ab | CST | WB | 1:1000 |
| 15 | p44/42 MAPK (Erk1/2) Ab | CST | WB | 1:1000 |
| 16 | SAPK/JNK Ab | CST | WB | 1:1000 |
| 17 | NF-κB p65 Ab | CST | WB | 1:1000 |
| 18 | p-p38 MAPK Ab | CST | WB | 1:1000 |
| 19 | p-p44/42 MAPK (Erk1/2) Ab | CST | WB | 1:2000 |
| 20 | p-SAPK/JNK Ab | CST | WB | 1:1000 |
| 21 | p-NF-κB p65 Ab | CST | WB | 1:1000 |
| 22 | GAPDH Ab | Sangon | WB | 1:5000 |
| 23 | IRDye® goat anti-rabbit IgG | LI-COR | WB | 1:10000 |
| 24 | recombinant PD-L1 protein | R&D | ELISA | 10 ug/ml |
| 25 | IFN-γ | Sino | ELISA&IF | 100 U/mL |
| 26 | sCD40L | Sino | ELISA | 100 ng/mL |
| 27 | SB203580 (p38 inhibitor) | MCE | ELISA | 30 uM |
| 28 | PD98059 (ERK1/2 inhibitor) | MCE | ELISA | 30 uM |
| 29 | SP600125 (JNK inhibitor) | MCE | ELISA | 30 uM |
| 30 | PDTC (NF-κB inhibitor) | MCE | ELISA | 100 uM |
| 31 | CD40 siRNA | GeneBio | RT-qPCR | 50 nM |

Note. All the reagents were purchased through the official website and stored in accordance with the instructions.

**SUPPLEMENTARY FIGURES and LEGENDS**

**Figure S1. (A)** Lymphocytes isolated from peripheral venous blood of TAO patients and negative controls were enriched and activated by anti-human CD3/CD28 monoclonal antibody beads, and the proportion of CD3+CD25+ cells were determined by FCM daily for the first 4 days of culture. **(B)** Activated T cells from TAO patients and negative controls were subjected to analysis of PD-1 expression by FCM. **(C)** T cells were treated with either PBS, recombinant human PD-L1 protein (10 ug/mL), PD-L1 combined with goat anti-human IgG (10 ug/mL), or PD-L1 combined with PD-L1 neutralizing antibody (10 ug/mL). And the supernatants were subjected to analysis of TNF-α and IL-2 content by ELISA after a 72-hour culture.

**Figure S2.** **(A)** The appearance and activity of OFs would change gradually in the process of OFs culturing, and part of OFs began to lose activity on generation six. **(B-C)** OFs from TAO patients and negative controls were co-cultured with autologous T cells (OFs : T cells = 1 : 10) for 48 hours, and the supernatant of cell culture were subjected to ELISA for assaying the distinction of sICAM-1, IL-6, IL-8, and HA expressions. **(D)** TAO-OFs were stimulated with PBS, PD-L1 (10 ug/mL), autologous T cells (OFs : T cells = 1 : 10), and PD-L1 combined with T cells for 48 hours, respectively. And the supernatants were subjected to analysis of CCL2 expression by ELISA. **(E)** TAO-OFs were treated with SB203580 (30 uM), PD98059 (30 uM), SP600125 (30 uM), and PDTC (100 uM) for 30 minutes, respectively, and then co-cultured with autologous T cells (OFs : T cells = 1 : 10) for 24 hours or not. The supernatants were subjected to analysis of CCL2 expression by ELISA.

**Figure S3. (A)** FCM of TAO-OFs compared to NC-OFs demonstrated differential expressions of CD40. **(B-C)** TAO-OFs transfected with CD40 siRNA or NC siRNA, and then co-cultured with autologous T cells, and the TAO-OFs were subjected to analysis of CD40 mRNA (**B**) by qPCR after a 48-hour culture and CD40 protein (**C**) by FCM after a 72-hour culture.
