# Supplementary figures and images for: PD-L1 Inhibits T Cell-Induced Cytokines and Hyaluronan Expression via the CD40-CD40L Pathway in Orbital Fibroblasts From Patients With Thyroid Associated Ophthalmopathy
Source: Front Immunol. 2022 May 10;13:849480. doi: 10.3389/fimmu.2022.849480 (PMC9128409; doi:10.3389/fimmu.2022.849480)

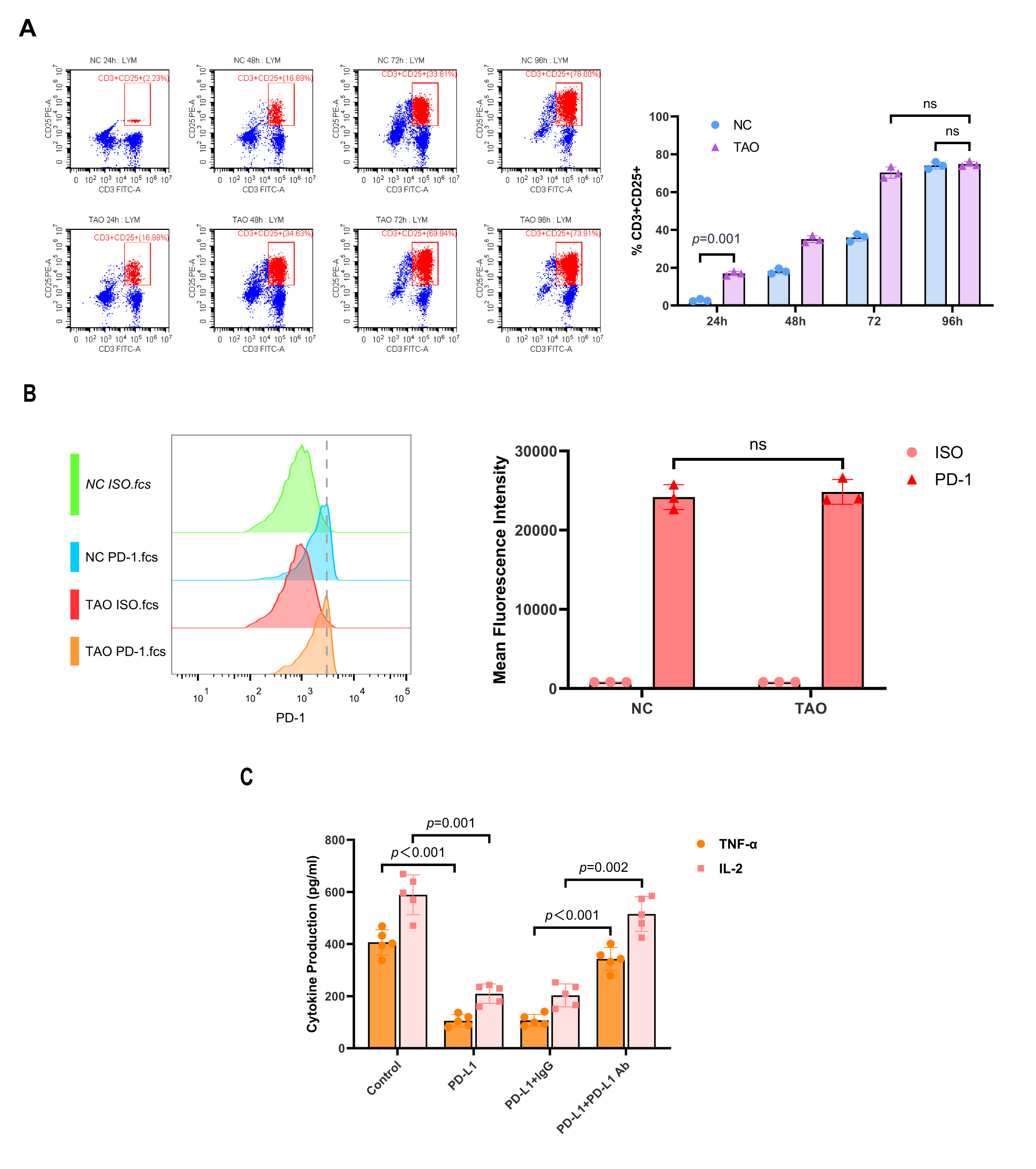

Supplement: Supplementary file 2 [file Image_1.tif]

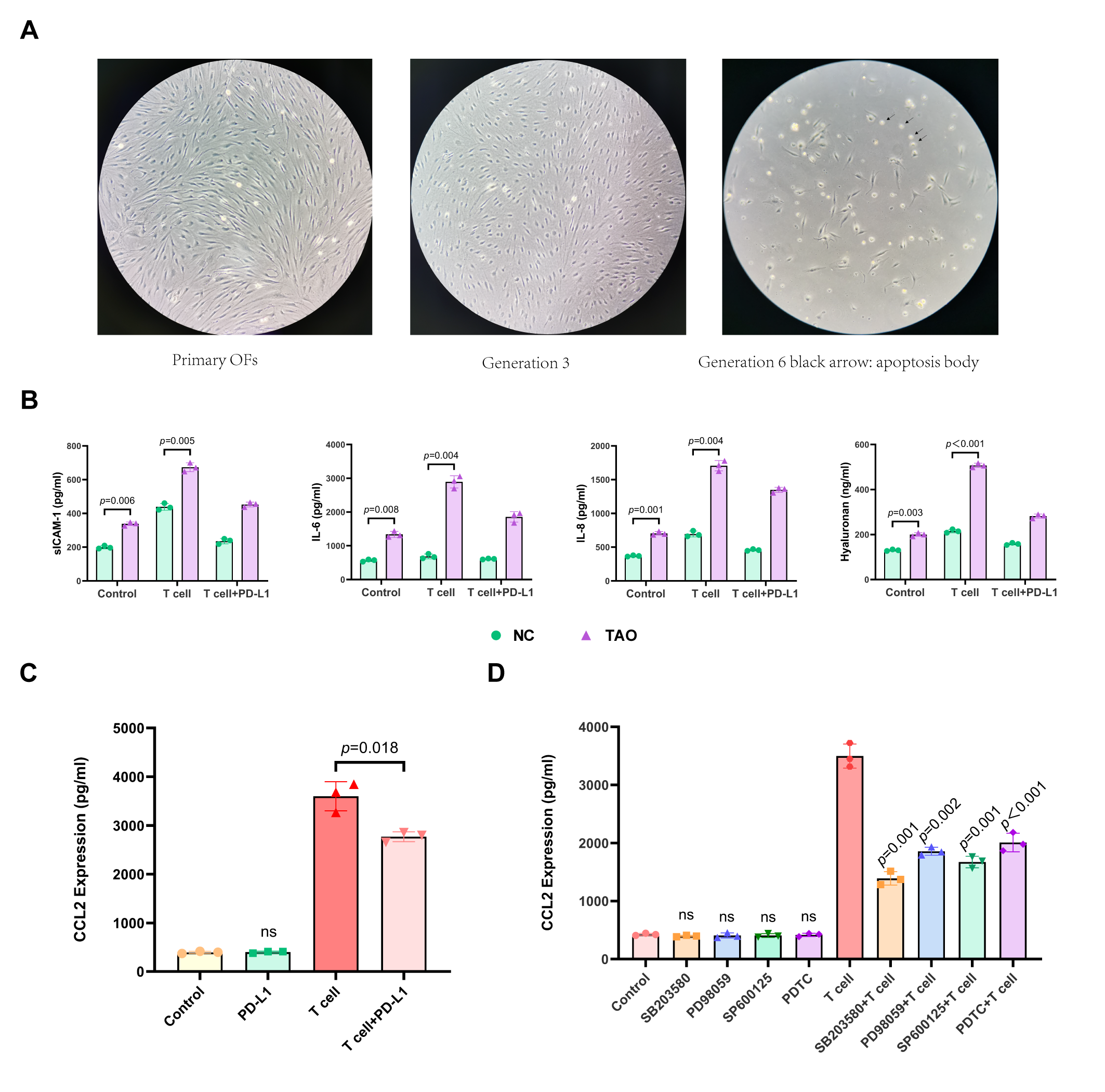

Supplement: Supplementary file 3 [file Image_2.tif]

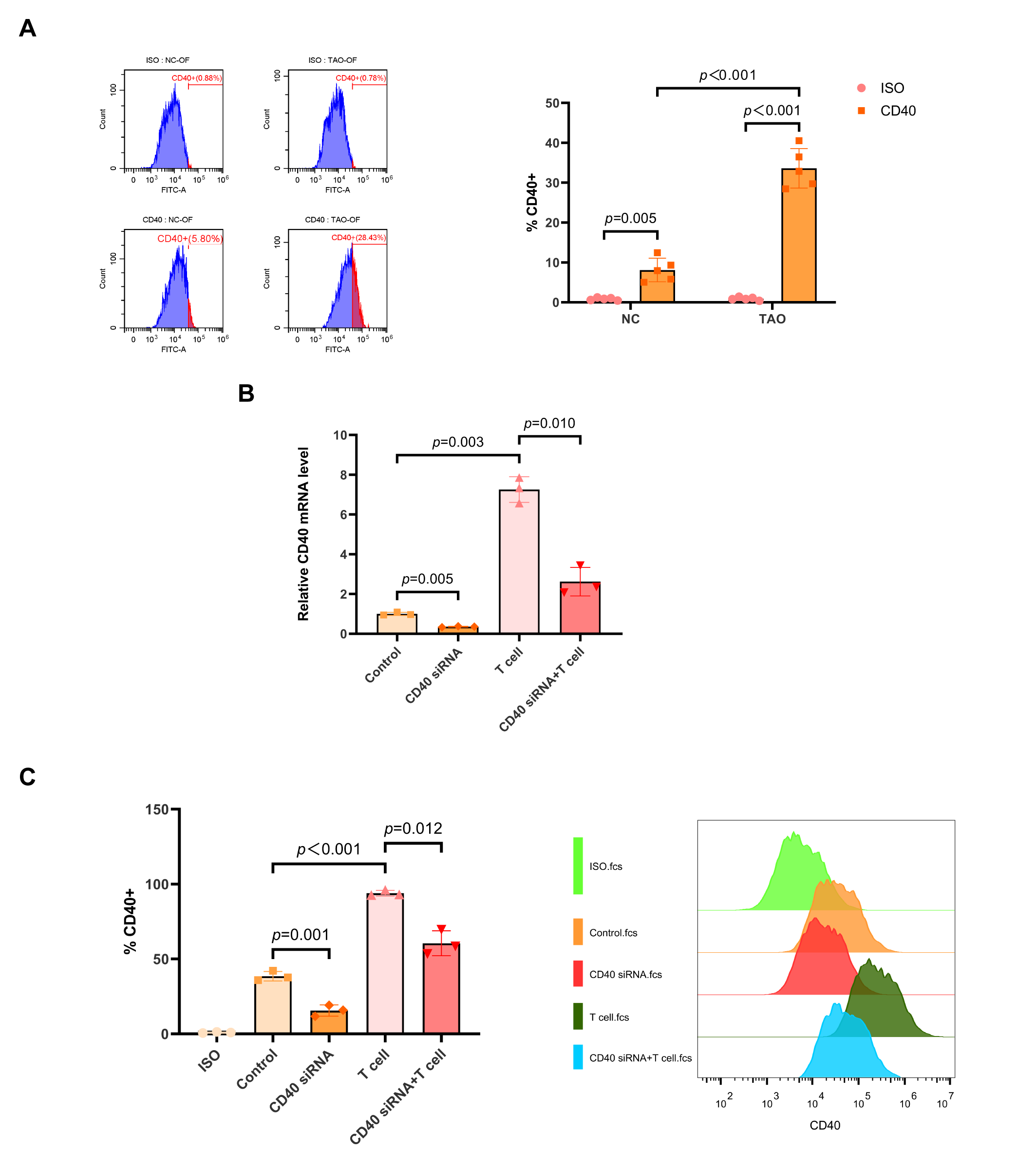

Supplement: Supplementary file 4 [file Image_3.tif]
